# Urinary Tuberculosis

**Published:** 1899-02-25

**Authors:** 


### URINARY TUBERCULOSIS.

Mr. Hurry Fenwick relates[1] the result of some very useful observations which he has made as to the contour and consistence of a thousand prostate glands as felt *per rectum*. He points out that few of us really know anything of the consistence and contour of the prostate in health, and moreover that the gland may differ most remarkably in size in the course of a few days and even of a few hours. In examining a prostate or bladder base the patient should first be made to empty his bladder as far as he can, and then bend over a couch or stool so that his body forms a right angle with his legs. In the case of a healthy young male the finger on entering the bowel recognises that the outlines of the two lobes of the prostate are equal, that their consistence is firmish, that their contour is a little prominent, and that they are separated by a fine groove—"the interlobar sulcus." "Accustom yourself to detect that sulcus, especially the upper part; it is the most important of urinary landmarks. Now pass your finger beyond the transverse line where the prostate merges into the bladder base. The bladder muscle is now felt smooth, soft, and even. You do not often feel the vesiculæ seminales mapped out, as you are led to expect; you rarely will. If you do there is a block at their orifices, or an inflammatory thickening, or a tuberculous deposit in their walls."

The most important disease which one may be led to suspect or recognise by rectal examination of the prostate and bladder base is urinary tuberculosis. No surgical disease is so likely to mislead by its vagaries, or to baffle well-meant efforts to relieve its active phases, or to so fiercely resent instrumental interference as urinary tuberculosis. Hence the importance of the earliest possible clue in its diagnosis, and this can be obtained in a large percentage of cases by examination of the testicles and of the bladder base. Urinary tubercle invades the urinary tract along certain regular highways. It commences most often in the epididymis. It is next found in the corresponding lobe of the prostate, next in the base and around the ureteral orifice of the corresponding side of the bladder, and, lastly, in the middle and lower third of the corresponding kidney near the pelvis. "Thus the 'rule of the road' is that a right epididymal knot leads to a right prostatic knot, to a loss of surface around the right ureteral orifice; and, finally, to a deposit in the right kidney." It is to be noted that a deposit, even though tuberculous, may absorb; but "no matter whether the deposit absorbs in one place, the wave travels persistently on to implicate another area, and it usually keeps to the side it started on. . . . If a patient who has no venereal history, who has never had any difficulty in micturition, and who has never had any urethral instrumentation, complains of pain in one kidney, and you find an indolent knot in the epididymis, or a buried knot in the prostatic lobe of that side, you diagnose tubercle of the kidney, and seek confirmation of this in the urine." Sometimes, however (in 18 cases out of 100), the tubercle appeared not to follow this rule, but to cross over to the other side. As showing the importance of examining the testis and the bladder base before using any instruments or giving a diagnosis, one must refer to the evil consequences which are likely to follow from instrumental interference in cases of tuberculosis. "Washing out the bladder daily with any form of sterile fluid, be it water, boric acid, or iodoform wash, will relieve in the early stages, but the patient pays dearly for his relief. You cannot fail to introduce septic matter, and the condition for the colonisation of the microbes you introduce or awake to activity are most favourable—a congested or ulcerated mucous membrane very susceptible to invasion, a neutral or faintly acid urine, and a very small amount of residual urine. I say definitely, after fifteen years' study, a patient with a tuberculous bladder, who is washed out as a matter of routine, has his kidney destroyed sooner and more thoroughly than he whose bladder is left alone." We should add that the condition described in most of the illustrative cases is one of unilateral swelling of, or nodule in, a prostatic lobe or seminal vesicle, generally together with deposit in the epididymis.

[1] Brit. Med. Jour., Feb. 18.

